# Putative enhancer sites in the bovine genome are enriched with variants affecting complex traits

**DOI:** 10.1186/s12711-017-0331-4

**Published:** 2017-07-06

**Authors:** Min Wang, Timothy P. Hancock, Iona M. MacLeod, Jennie E. Pryce, Benjamin G. Cocks, Benjamin J. Hayes

**Affiliations:** 1 School of Applied Systems Biology, La Trobe University, Bundoora, VIC 3083 Australia; 2 Agriculture Victoria, AgriBio, Centre for AgriBioscience, Bundoora, VIC 3083 Australia

## Abstract

**Background:**

Enhancers are non-coding DNA sequences, which when they are bound by specific proteins increase the level of gene transcription. Enhancers activate unique gene expression patterns within cells of different types or under different conditions. Enhancers are key contributors to gene regulation, and causative variants that affect quantitative traits in humans and mice have been located in enhancer regions. However, in the bovine genome, enhancers as well as other regulatory elements are not yet well defined. In this paper, we sought to improve the annotation of bovine enhancer regions by using publicly available mammalian enhancer information. To test if the identified putative bovine enhancer regions are enriched with functional variants that affect milk production traits, we performed genome-wide association studies using imputed whole-genome sequence data followed by meta-analysis and enrichment analysis.

**Results:**

We produced a library of candidate bovine enhancer regions by using publicly available bovine ChIP-Seq enhancer data in combination with enhancer data that were identified based on sequence homology with human and mouse enhancer databases. We found that imputed whole-genome sequence variants associated with milk production traits in 16,581 dairy cattle were enriched with enhancer regions that were marked by bovine-liver H3K4me3 and H3K27ac histone modifications from both permutation tests and gene set enrichment analysis. Enhancer regions that were identified based on sequence homology with human and mouse enhancer regions were not as strongly enriched with trait-associated sequence variants as the bovine ChIP-Seq candidate enhancer regions. The bovine ChIP-Seq enriched enhancer regions were located near genes and quantitative trait loci that are associated with pregnancy, growth, disease resistance, meat quality and quantity, and milk quality and quantity traits in dairy and beef cattle.

**Conclusions:**

Our results suggest that sequence variants within enhancer regions that are located in bovine non-coding genomic regions contribute to the variation in complex traits. The level of enrichment was higher in bovine-specific enhancer regions that were identified by detecting histone modifications H3K4me3 and H3K27ac in bovine liver tissues than in enhancer regions identified by sequence homology with human and mouse data. These results highlight the need to use bovine-specific experimental data for the identification of enhancer regions.

**Electronic supplementary material:**

The online version of this article (doi:10.1186/s12711-017-0331-4) contains supplementary material, which is available to authorized users.

## Background

Genomic selection is a powerful tool that has rapidly accelerated genetic gains in the dairy industry [1]. Genomic estimated breeding values (GEBV) for ranking selection candidates are calculated as the sum of the individual effects of genome-wide single nucleotide polymorphisms (SNPs). Genomic prediction for a given trait of interest would be most accurate if all causative variants that affect a trait were known and used in the prediction. For most complex traits, such as milk production in dairy cattle, very few causal variants are known [2] and therefore it is unlikely that the full set of causative variants are contained within the SNP panels used for routine evaluation. The task of identifying causative variants for complex traits is challenging since it is likely that a very large number of causative mutations with small effects contribute to the total genomic variation of the trait [3].

Recent research has indicated that much of the variation that affects complex traits lies in the non-coding genome [2], particularly transcriptional regulatory elements. Enhancers, which are also called locus control regions (LCR) or upstream activating sequences (UAS) [4], are non-coding DNA sequences, which when they are bound by specific proteins, enhance the transcriptional level of a related gene or set of genes [4]. To date, the identification of genomic regulatory elements including enhancers has followed two main approaches. Firstly, evolutionarily conserved non-coding sequences between mammalian species or higher vertebrates [5, 6] have been used to identify the more conserved developmental enhancers [7, 8]. Secondly, a more recent approach that uses chromatin immunoprecipitation followed by high-throughput sequencing (ChIP-Seq) can detect candidate enhancers on a genome-wide scale. This approach uses antibodies to snapshot transcriptional proteins that are bound to DNA sequences in vivo, and has revealed a much larger number of candidate enhancers [4, 9] than the previous approach, the majority of which were detected only in a specific physiological context [10]. Examples of biological signals that allow the identification of enhancers are mono-methylation of lysine 4 on histone H3 (H3K4me1) [11–13], p300-CBP coactivator protein family [14–16], tri-methylation of lysine 4 on histone H3 (H3K4me3) and acetylated lysine 27 on histone H3 (H3K27ac) [17–19]. The histone mark H3K4me3 displays a bimodal distribution that flanks the transcription start sites (TSS) of active or to be expressed genes in eukaryotes [20]. It is a prevalent histone mark for promoters [21, 22] and is also found in the coding regions of genes [21, 22], and occasionally it marks active enhancers [13, 20, 23, 24]. The histone mark H3K27ac distinguishes active enhancers from poised enhancers at a tissue-specific level and in a developmental-specific manner [12, 25]. It also marks active promoters [12] and displays broader profiles than H3K4me3, which is in line with its association with open chromatin [12, 13]. The number of histone marks and co-occupation of other cellular elements collaboratively define the transcriptional state of a genomic region [20].

The aim of this study was to identify bovine enhancer regions in silico based on sequence homology with functional annotation data in other species in addition to bovine ChIP-Seq data. We evaluated the influence of mutations in enhancer regions on complex production traits by performing a multi-breed genome-wide association study (GWAS) with imputed whole-genome sequence data in 16,581 cattle followed by meta-analysis and enrichment analysis.

## Methods

### Mammalian enhancer sets

We used four public mammalian enhancer datasets to search for bovine putative enhancers, i.e. VISTA [26], FANTOM5 [27], dbSUPER [28] and the Villar et al. [29] study. The VISTA enhancer browser [26] selects evolutionarily ultra-conserved sequences between vertebrates and validates enhancer activities in transgenic mouse reporter assays [6]. The functional annotation of the mammalian genome 5 project (FANTOM5) [27] provides a repository of active enhancers from various human and mouse tissues. FANTOM5 enhancers are defined by bidirectional transcription signals at the 5′ end of the transcription start site (TSS) using single-molecule HeliScope cap analysis of gene expression (CAGE) [30]. The database dbSUPER collects data on super-enhancers from various human or mouse tissues across multiple studies [28]. A super-enhancer (also known as a stretch enhancer) is a group of active enhancers that are densely clustered in a 10 to 30-kb region and are highly associated with cell identity genes and disease-associated genomic variations [31]. We combined these sets of homologous enhancers with predicted bovine enhancers from Villar et al. [29] who used ChIP-Seq to detect, in the bovine genome, binding sites to H3K4me3 and H3K27ac [17–19] in the liver tissue of four bulls. Finally, the library of enhancers that we used to identify bovine putative enhancers includes 4481 VISTA (2235 human and 2246 mouse), 109,882 FANTOM5 (65,423 human and 44,459 mouse), 1745 dbSUPER (607 human and 1138 mouse), 13,797 Villar H3K4me3 (13,797 bull) and 45,784 Villar H3K27ac (45,784 bull) sequences, which were downloaded from their respective host sites on 10 August 2015, 6 October 2015, 14 September 2015, 4 September 2015 and 4 September 2015.

### Genotypes

Illumina BovineHD BeadChip 800 K SNPs (real or imputed) were available for 3311 Holstein bulls, 8478 Holstein cows, 875 Jersey bulls and 3917 Jersey cows. Among these individuals, 145 Holstein bulls and 47 Jersey bulls were from the 1000 Bull Genomes Project [32], and most of the cows were from the 10,000 Holstein Cow Genomes Project and Jer-nomics Project [33]. Quality control and imputation were performed as described in [34], with an additional filter to retain only the SNPs that overlapped with sequence variants discovered in the 1000 Bull Genomes Project (run 4). The genotypes of all animals were then imputed to whole-genome sequence (WGS) using Fimpute [35] with a reference population of 1147 individuals with whole-genome sequences from the 1000 Bull Genomes Project (run 4). After imputation, 28,899,038 WGS variants were available. All genomic loci were mapped to the bovine genome assembly UMD3.1 (bostau6) [36].

### Phenotypes

Phenotypes for the genotyped animals were available for milk production traits including fat yield (FY), milk yield (MY) and protein yield (PY) from the national dairy database operated by DataGene (Melbourne, Australia). The phenotypes used in the analyses were trait deviations (TD) for cows and daughter trait deviations (DTD) for bulls. TD were calculated based on cows’ lactation records (three lactations on average) and corrected for known fixed effects as per DataGene routine evaluations from the April 2013 official breeding value run. DTD were generated from nationwide progeny test data collected on many bulls’ daughters, and were corrected for known fixed effects such as herd, year and season. The animals used in our study were the same as or overlapped with those in previous publications [34, 37, 38].

### Other data

The reference genomes used in this analysis, GRCh38.p4 (hg38), GRCm38.p4 (mm10) and UMD3.1.1 (bostau8), along with their annotation files, were downloaded from the National Centre for Biotechnology Information (NCBI) Reference Sequence Database (RefSeq) [39] on 20 August 2015. Genomic coordinate conversion files (chain file format) were downloaded from the UCSC (University of California, Santa Cruz) database [40] on 29 February 2016. Annotations for the sequence variants were collated using NGS-SNP [41]. The bovine quantitative trait loci (QTL) annotation file was downloaded from the Animal QTL database (Animal Qtldb) [42] on 17 May 2016.

### Mapping bovine candidate enhancers

The human and mouse enhancer regions from VISTA, FANTOM5 and dbSUPER were mapped to the bovine reference genome assembly UMD3.1.1 via command line applications Nucleotide Basic Local Alignment Search Tool (BLASTn) [43] (default settings except for the e-value were $4 \times 10^{ - 17}$) and UCSC Batch Coordinate Conversion (liftOver) [40] (default settings), respectively. The BLASTn approach measures local sequence similarity to identify which query segments can be matched to different parts of the target genome [43]. The liftOver approach measures global sequence similarity where the query sequence is optimised to the best matching location in the target genome, although the best matching location may be stretched out on a much longer region than the query sequence [40]. The BLASTn software returned specific genomic coordinates for mapped query segments, whereas the liftOver command application returned a mapped file for all the genomic coordinates that were found in the target genome, and an unmapped file for all the query sequences that were partially or fully unmapped. We considered all the returned queries with full or partial hits as mapped input sequences in BLASTn, and all the queries that were not marked as fully unmapped were considered as mapped input sequences in liftOver. LiftOver outputs were combined with BLASTn results. All regions from the combined set that overlapped over more than one bp were merged into a longer and non-overlapping genomic interval. Bovine enhancer data from ChIP-Seq H3K4me3 and H3K27ac signals [29] were directly merged into the non-overlapping set, respectively.

### Genome-wide association study

A multi-breed genome-wide association study (GWAS) was performed to detect imputed WGS variants that were associated with FY, MY and PY. Following the approach described by [37], Holstein and Jersey data were combined, but the analyses were separated by gender, because phenotype measurements in bulls and cows have different degrees of uncertainty [34]. The efficient mixed-model association expedited (EMMAX) analysis software package [44] was used to fit the 28,899,038 WGS variants one by one in the linear mixed model:

$${\mathbf{Y}} = {\mathbf{W}}{\varvec{\upomega }} + {\mathbf{X}}{\varvec{\upbeta }} + {\mathbf{Zu}} + {\mathbf{e}},$$

where ${\mathbf{Y}}$ is a vector of phenotypes (DTD for bulls and TD for cows); ${\mathbf{W}}$ is the design matrix that allocates phenotypes to fixed effects accounting for overall mean and breeds; ${\varvec{\upomega}}$ is a vector of fixed effect solutions; ${\mathbf{X}}$ is a vector of animal genotypes; ${\varvec{\upbeta}}$ is a vector of genotype effects; ${\mathbf{Z}}$ is a matrix that allocates phenotypic records to animals and ${\varvec{\upmu}}$ is a vector of polygenic breeding values fitted as a random effect and assumed to follow a normal distribution $N\left( {{\varvec{0}}, {\mathbf{G}}\sigma_{g}^{2} } \right)$, where $\sigma_{g}^{2}$ is the genetic variance of the trait, and ${\mathbf{G}}$ is the genomic relationship matrix calculated from the 800 K genotypes as in [45]; and ${\mathbf{e}}$ is a vector of residual errors distributed $N\left( {{\varvec{0}}, {\mathbf{I}}\sigma_{e}^{2} } \right)$, where $\sigma_{e}^{ 2}$ is the error variance. The polygenic breeding values were included in the model to avoid false positive SNP effects due to population structure and sub-structure [44].

### Meta-analysis

The multi-breed GWAS results from bull and cows were combined using an inverse-variance weighting meta-analysis within a fixed effect model as described by [46]. We did not perform a joint analysis since inclusion of different accuracies for the phenotypes of bulls and cows was not possible in EMMAX. For the inverse-variance weighted meta-analysis, the following were calculated:

1. The standard error of SNP effects is calculated as follows:
  1 $${\text{se}}_{i,j} = \left| {\frac{{\beta_{i,j} }}{{Q\left( {p_{i,j} } \right)}}} \right|,$$
  where $i$ indicates the SNP at position $i$, $j$ indicates the gender cohort, ${\text{se}}$ is the standard error of the SNP effect, $\beta$ is the SNP effect output from EMMAX, $Q$ is the quantile function of the standard normal distribution and $p$ is the GWAS *P* value output from EMMAX.
2. The inverse-variance weight for each SNP is then calculated as:
  2 $$w_{i,j} = \frac{1}{{{\text{se}}_{i,j} }},$$
  where $i$ and $j$ are as defined above, $w$ is the inverse-variance weight, and ${\text{se}}$ is the standard error of the SNP effect calculated from Eq. (1).
3. The inverse-variance weighted effect for each SNP is then calculated as:
  3 $$\hat{\beta }_{i,j} = \beta_{i,j} \times w_{i,j} ,$$
  where $i$ and $j$ are as defined above, $\hat{\beta }$ is the weighted effect, $\beta$ is the SNP effect output from EMMAX.
4. The SNP effect from the meta-analysis that combines gender cohorts is calculated as:
  4 $$\tilde{\beta }_{i} = \frac{{\mathop \sum \nolimits_{i \in j} \hat{\beta }_{i,j} }}{{\mathop \sum \nolimits_{i \in j} w_{i,j} }},$$
  where $i$ and $j$ are as defined above, $\tilde{\beta }$ is the SNP effect from the meta-analysis, $\hat{\beta }$ is the weighted effect calculated from Eq. (3), and $w$ is the weight calculated from Eq. (2).
5. The variance of the SNP effect from the meta-analysis that combines gender cohorts is calculated as:
  5 $$\tilde{v}_{i} = \sqrt {\frac{n}{{\mathop \sum \nolimits_{i \in j} w_{i,j} }}} ,$$
  where $i$ is as above, $\tilde{v}$ is the variance of the SNP effect from the meta-analysis, $n$ is the number of cohorts being combined in the meta-analysis (here, $n$ is equal to 2 because the bull and cow cohorts were combined), and $w$ is the weight calculated from Eq. (2).
6. The P value from the meta-analysis that combines gender cohorts is calculated as:
  6 $$\tilde{p}_{i} = 2 \times \left( {1 - F\left( {\left| {\frac{{\tilde{\beta }_{i} }}{{\tilde{v}_{i} }}} \right|} \right)} \right),$$
  where $i$ is as above, $\tilde{p}$ is the P value output from the meta-analysis, $F$ is the quantile function of the standard normal distribution, $\tilde{\beta }$ is the SNP effect from the meta-analysis calculated from Eq. (4), and $\tilde{v}$ is the variance of the SNP effect from the meta-analysis calculated from Eq. (5).

Variants with no effect or with a P value of 1 were removed from the downstream analysis. Of the 28,899,038 imputed WGS variants input for FY cohort, after the meta-analysis, 23,462,606 variants remained. Of the 28,899,038 imputed WGS variants input for MY cohort, after the meta-analysis 23,462,606 variants remained. Of the 28,899,038 imputed WGS variants input for PY cohort, after the meta-analysis 23,470,573 variants remained. Significant variants from the meta-analysis were selected using the same threshold as in the GWAS $\left( {P \le 10^{ - 8} } \right)$.

### Enrichment analysis

The bovine candidate enhancers were categorised into five enhancer sets based on their input databases: VISTA, FANTOM5, dbSUPER, Villar H3K4me3 or Villar H3K27ac. Two enrichment analyses, i.e. permutation test and gene set enrichment analysis (GSEA), were performed to examine if any of the bovine candidate enhancer sets were enriched with variants associated with FY, MY or PY. The permutation test compared the number of significant SNPs in an enhancer set with the null distribution sampled from the rest of the genome. However, the need for a predefined threshold for statistical significance in the permutation tests may result in not detecting relevant biological differences that are modest relative to the noise that is inherent to the data [47]. This insensitivity of the permutation test was partly overcome by GSEA, which considered the distribution of all effects and tested if SNPs in an enhancer set were responsible for the enrichment signal, without applying any significant threshold [47].

The permutation test was run for 10,000 random repeats to test if the number of significant SNPs in an enhancer set was significantly larger than that obtained by random chance. The numbers of SNPs and of significant SNPs in an enhancer set, and the number of SNPs in a random draw are denoted as $N_{E}$, $n_{s}$ and $m_{s}$, respectively. In each permutation, a significant SNP was determined by a global P value cut-off of $P \le 10^{ - 8}$. The fold change of the enrichment was defined as the ratio of $n_{s}$ to the mean of all $m_{s}$ in random samples. The ranking position of $n_{s}$ within the distribution of all $m_{s}$ over all random samples, denoted as $R$, was determined, and a P value to test the significance of the ranking was computed. For the largest $n_{s}$ among all $m_{s}$, the P value was set to <0.0001 and otherwise it was $\frac{R}{10001}$. Our permutation tests resulted in 15 independent analyses (3 phenotypes $\times$ 5 enhancer databases).

The GSEA statistics was the cumulative sum of the effects of SNPs in putative enhancers computed from the sorted list of all SNP effects. Here, the effect was assessed by –log10 (P value). At each point in the GSEA algorithm, the test statistic $ES$ was computed as follows:

$$P_{hit} \left( {E_{i} , j} \right) = \mathop \sum \limits_{{\begin{array}{*{20}c} {v_{j} \in E_{i} } \\ {m \le j} \\ \end{array} }} \frac{{p_{m} }}{{N_{R} }},\quad where\;N_{R} = \mathop \sum \limits_{{v_{j} \in E_{i} }} p_{m} ,$$

$$P_{miss} \left( {E_{i} , j} \right) = \mathop \sum \limits_{{\begin{array}{*{20}c} {v_{j} \notin E_{i} } \\ {m \le j} \\ \end{array} }} \frac{1}{{\left( {N - N_{E} } \right)}},$$

$$ES = P_{hit} \left( {E_{i} , j} \right) - P_{miss} \left( {E_{i} , j} \right),$$

where $j$ is the position of the effect of an enhancer SNP in the sorted list of all SNP effects, $P_{hit}$ and $P_{miss}$ are respectively the cumulative probability of observing all enhancer SNPs and all non-enhancer SNPs up to position $j$, thus $ES$ denotes the level of enrichment of enhancer SNPs up to position $j$. The position at which $ES$ reaches the maximum deviation from 0, $ES\_max$, defines the strength of the enrichment signal in the enhancer set. All enhancer SNPs that are identified before $ES$ reaches $ES\_max$ are assigned to the candidate core enhancer set.

The significance of each GSEA was determined in a similar way as that for the permutation test described above. We randomly shuffled the SNPs within the sorted list while retaining the sorted positions of −log10 (P value) and recalculated the $ES\_max$ value. The shuffle was repeated 10,000 times and 10,000 $ES_{NULL}$ values were obtained. A GSEA result was considered significant if the $ES\_max$ value was larger than all $ES_{NULL}$ values. Our GSEA resulted in 15 independent analyses (3 phenotypes $\times$ 5 enhancer databases), but the sets of core enhancer SNPs were only those from the significant GSEA cohorts.

## Results

### Mapping bovine candidate enhancers

Two aligners, BLASTn and liftOver, were used to map human and mouse enhancers on the bovine genome (Table 1). All sets of bovine putative enhancer regions covered bovine chromosome 1 to 30. The bovine reference genome assembly bostau6 and bostau8 do not include chromosome Y. The mapping rate was defined as the ratio between the number of query sequences found in the bovine genome and the number of query sequences input for search. Cross-species mapping rates were equal to, in decreasing order, 96% for VISTA, 92% for dbSUPER and 87% for FANTOM5. The number of overlaps between BLASTn and liftOver results was small for FANTOM5 (<10%), moderate for dbSUPER (16%) and high for VISTA (71%). Over 93 and over 95% of the dbSUPER hits were within 10 and 30 kb to each other, respectively. As expected, homologous enhancer sequences were on average shorter than their respective query sequences (Table 1).

**Table 1:** Mapping of bovine candidate enhancers

db^a^	Query^b^			Met^f^	m%^g^	Hits^h^			eSNP^l^
	ISeq^c^	u (bp)^d^	σ (bp)^e^			OSeq^i^	µ (bp)^j^	σ (bp)^k^	
VISTA	4481	1959	1395	COM	4285 (96%)	9945	896	710	82,865
				LO	3808 (85%)	964	1399	883	
				BN	3627 (81%)	9945	896	710	
FANTOM5	109,882	277	158	COM	95,123 (87%)	30,371	231	115	50,447
				LO	94,302 (86%)	6061	245	503	
				BN	10,054 (9%)	30,389	231	115	
dbSUPER	1745	45,750	56,541	COM	1605 (92%)	50,938	739	763	282,285
				LO	1549 (88%)	32	1916	2447	
				BN	1113 (64%)	50,938	739	763	
H3K4me3	13,797	2393	879	NA	13,797 (100%)	13,660	2394	879	302,659
H3K27ac	45,784	2304	1910	NA	45,784 (100%)	42,963	2305	1910	965,716

^a^Database from which input query sequences were obtained

^b^Query sequence downloaded from respective host sites (may include overlapping regions)

^c^Number of input enhancer query sequences

^d^Mean length of input enhancer query sequences (measured in bp)

^e^Standard deviation length of input enhancer query sequences (measured in bp)

^f^Method that returned values in column m%; COM: a set of non-overlapping regions from the combined results of liftOver and BLASTn; LO: liftOver; BN: BLASTn

^g^Number of mapped query sequences (ratio of mapped query sequences); *NA* not applicable

^h^Hit is a non-overlapping genomic interval in the bovine genome that matches with at least one query sequence from the respective input database

^i^Number of non-overlapping candidate bovine enhancer genomic intervals; all hits, i.e. output from the respective software were merged into non-overlapping genomic intervals; some OSeq values were larger than corresponding ISeq values because one query sequence was found at multiple locations in the bovine genome

^j^Mean length of bovine putative enhancer sequences (measured in bp)

^k^Standard deviation length of bovine putative enhancer sequences (measured in bp)

^l^Number of imputed whole-genome sequence variants in OSeq genomic intervals

**Fig. 1:**
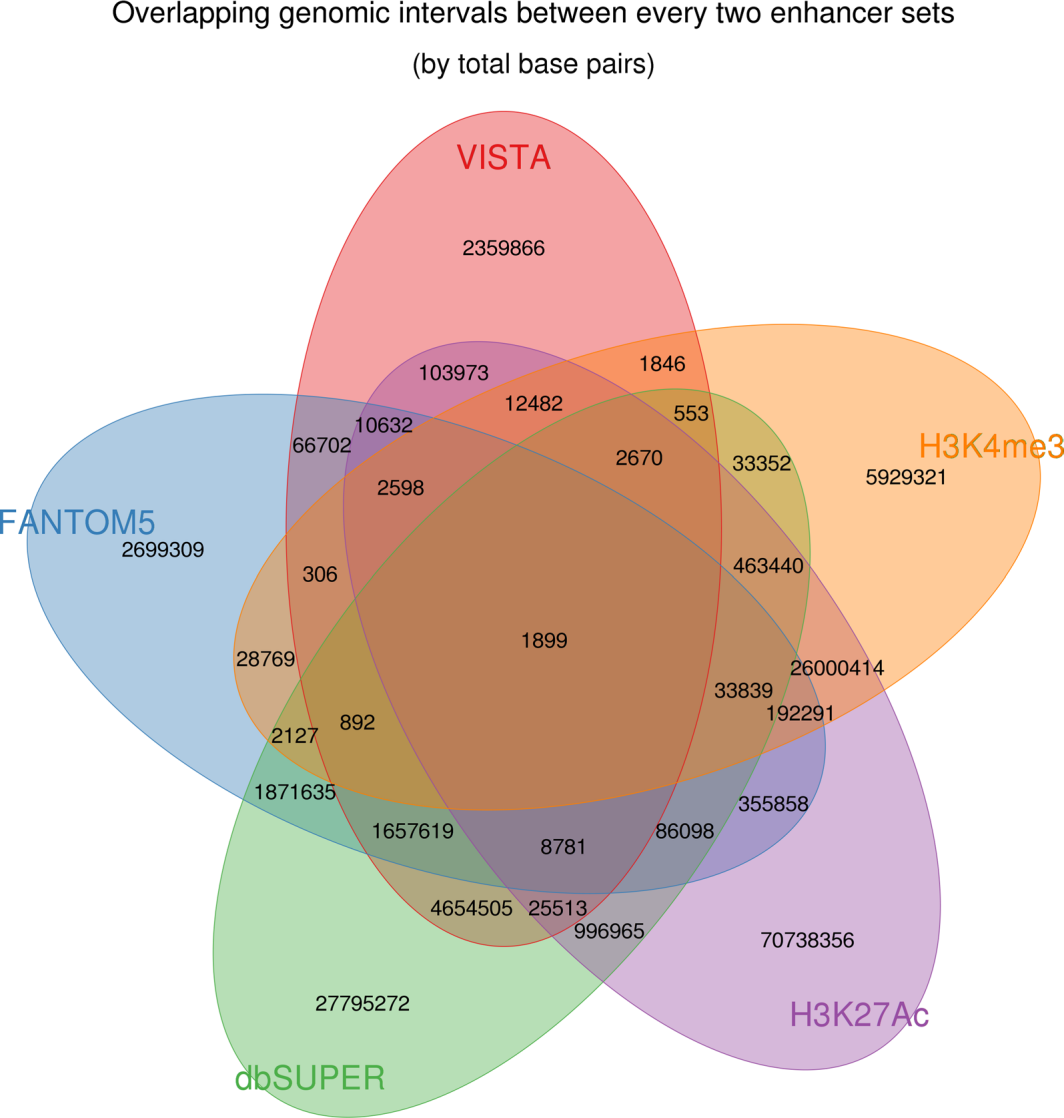
Degree of overlap between bovine enhancer sets (measured by total base pairs)

A pair-wise comparison was performed to examine the degree of overlap between the sets of bovine putative enhancer regions (Fig. 1). Villar H3K27ac and dbSUPER were the two major enhancer sets, because the Villar H3K27ac set covered 82% of the Villar H3K4me3 bovine genomic intervals, and the dbSUPER set covered 71% of the VISTA and 52% of the FANTOM5 bovine genomic intervals (Table 2; Fig. 1). However, the Villar H3K27ac and dbSUPER sets differed substantially (less than 5% overlaps; Table 2; Fig. 1).

**Table 2:** Degree of overlap between the sets of bovine enhancers analysed

	VISTA^a^ (%)	FANTOM5^b^ (%)	dbSUPER^c^ (%)	H3K27ac^d^ (%)	H3K4me3^e^ (%)
VISTA	100	19.63	71.29	1.89	0.26
FANTOM5	24.92	100	52.18	9.86	3.74
dbSUPER	16.88	9.73	100	4.30	1.43
H3K27ac	0.17	0.70	1.63	100	26.97
H3K4me3	0.07	0.80	1.65	81.66	100

Each value in the table represents the ratio, expressed as a percentage, of the total overlapping base pairs between the two enhancer sets listed in a row and column, relative to the total number of base pairs in the enhancer set listed in the corresponding row

^a^VISTA is a database for evolutionarily ultra-conserved sequences between vertebrates

^b^FANTOM5 is a database for active enhancers from various human and mouse tissue

^c^dDbSUPER is a database for super-enhancers from various human or mouse tissues across multiple studies

^d^H3K27ac represents the dataset from the Villar et al. [29] study, which used ChIP-Seq profiling to detect the regions of the bovine genome that contained the histone modification signal H3K27ac from four bulls’ liver tissues

^e^H3K4me3 represents the dataset from the Villar et al. [29] study which used ChIP-Seq profiling to detect the regions of the bovine genome that contained the histone modification signal H3K4me3 from four bull’s liver tissues

**Fig. 2:**
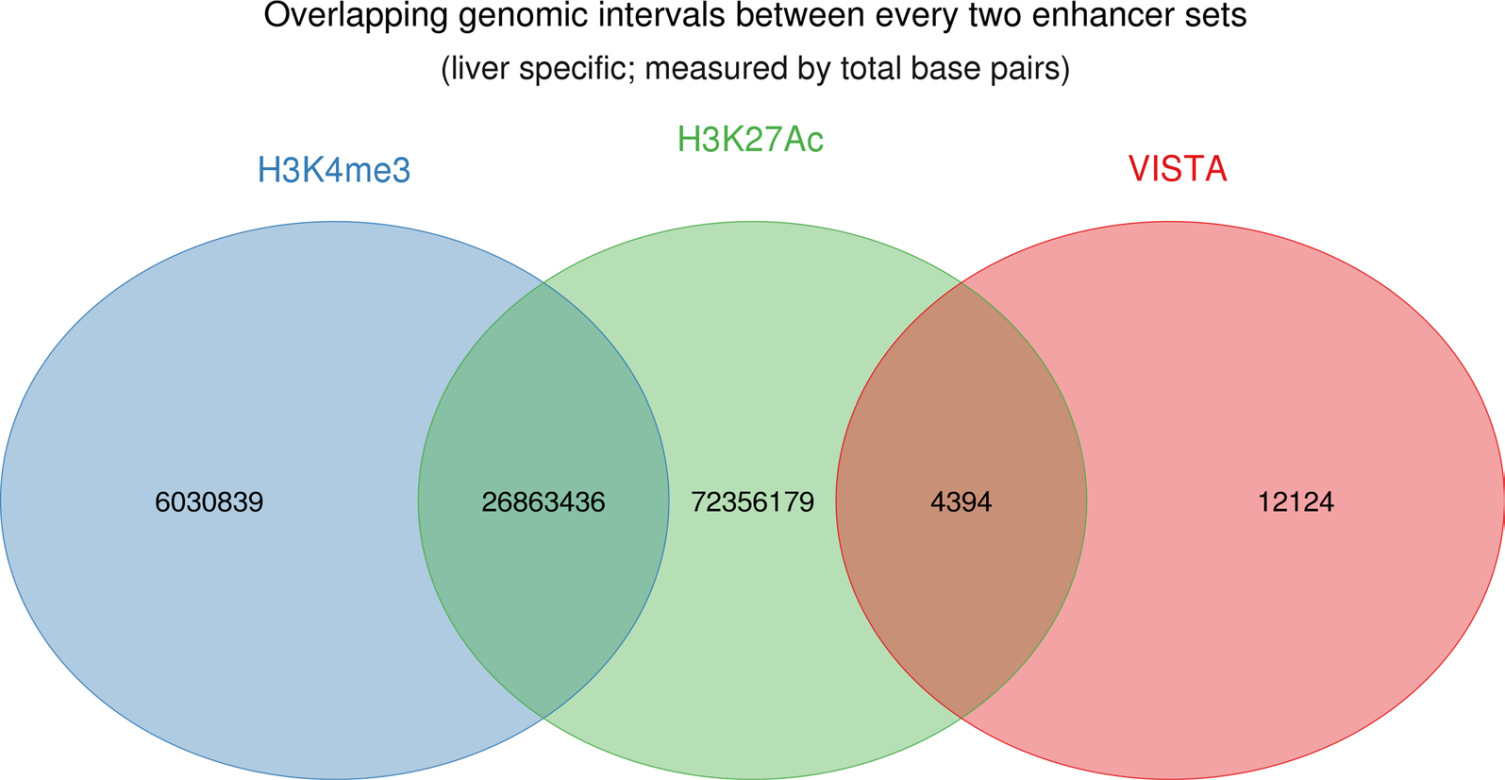
Degree of overlap between enhancer sets from liver (measured by total base pairs)

Given that enhancers are highly tissue-specific, we compared only liver-specific enhancers from homologous enhancer sets and Villar ChIP-Seq enhancer set. Only eight VISTA enhancers were liver-specific, which generated 236 bovine putative liver enhancers. We could not determine from which tissue FANTOM5 sequences originated. No liver data was available in the dbSUPER database. The bovine putative VISTA-liver enhancers overlapped very little with the bovine-liver H3K27ac (27%) and did not overlap at all with the bovine-liver H3K4me3 enhancers (Fig. 2).

### Genome-wide association study

**Fig. 3:**
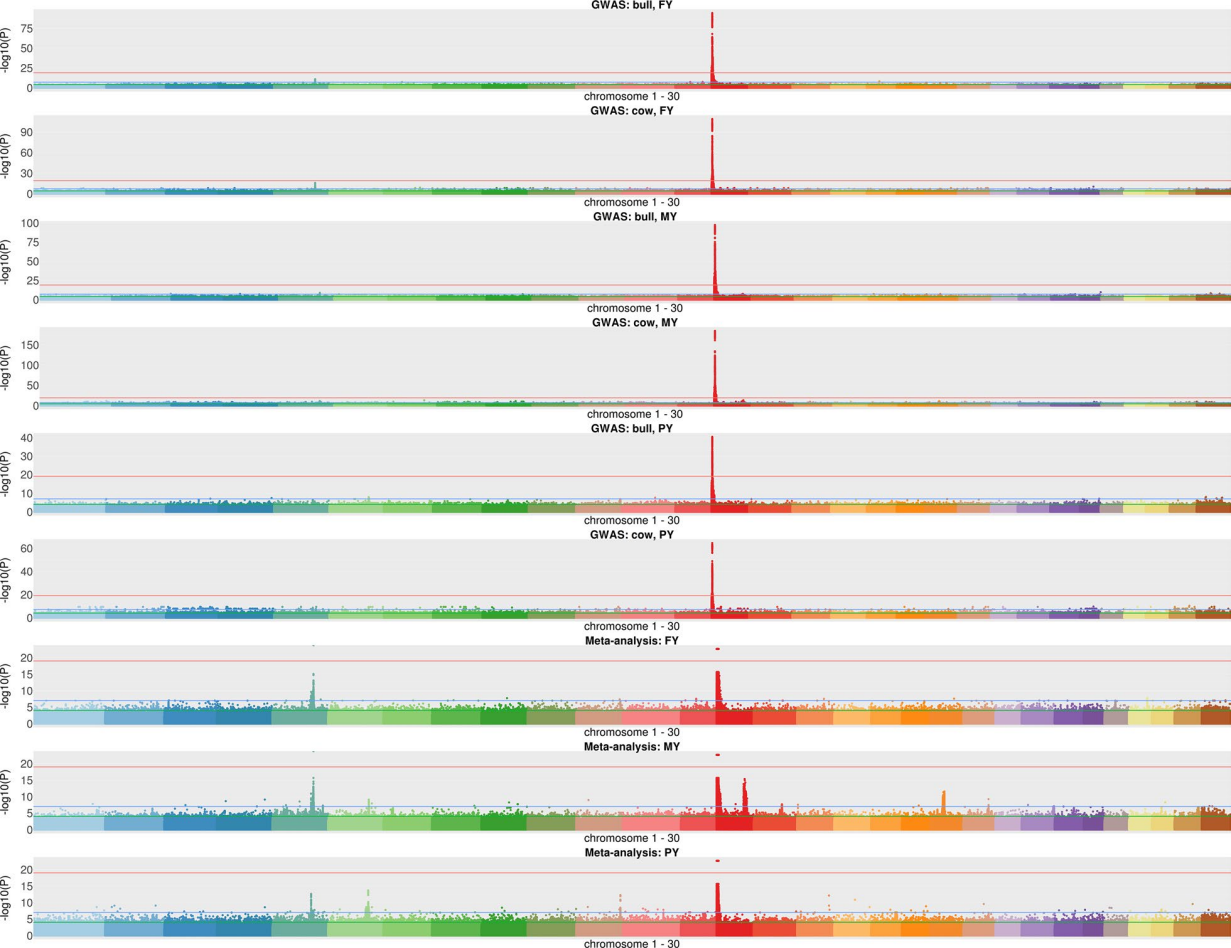
Manhattan plots: all GWAS and meta-analysis cohorts

The number of significant variants $\left( {P \le 10^{ - 8} } \right)$ for each trait is in Table 3. Bulls and cows demonstrated similar GWAS profiles for the respective phenotype cohorts (Fig. 3).

**Table 3:** Number of imputed whole-genome sequence variants and of significant variants $\left( {P \le 10^{ - 8} } \right)$

Phenotype	GWAS			Meta-analysis	
	Gender	Variants tested	Significant variants	Filtered variants tested	Significant variants
FY	Bulls	28,899,038	3720 (0.013%)	23,462,193	6967 (0.030%)
	Cows		3474 (0.012%)		
MY	Bulls		4408 (0.015%)	23,455,977	10,071 (0.043%)
	Cows		6801 (0.024%)		
PY	Bulls		1786 (0.006%)	23,470,099	4804 (0.020%)
	Cows		2981 (0.010%)

### Meta-analysis

**Fig. 4:**
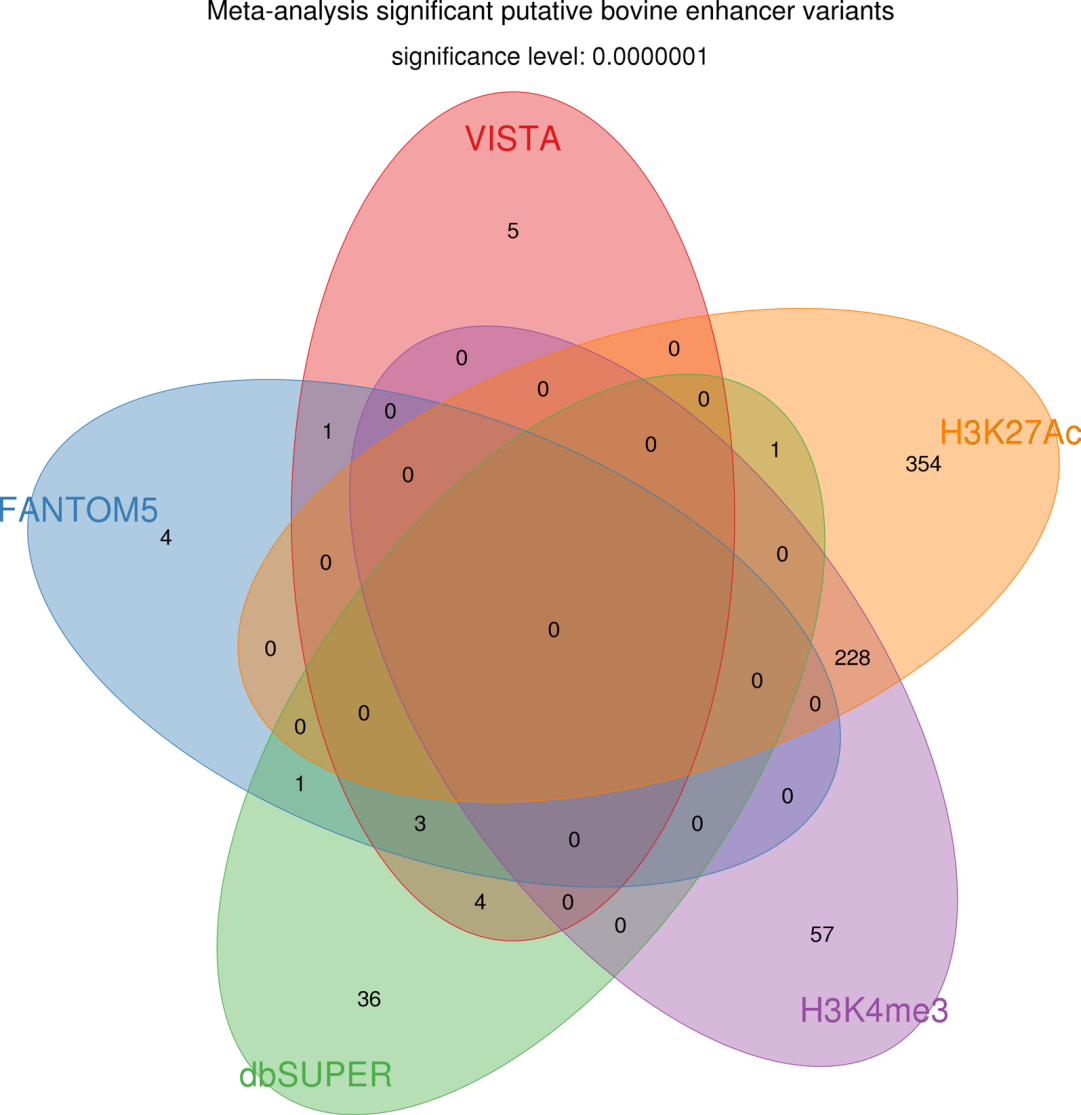
Degree of overlap between significant imputed WGS SNPs $\left( {P \le 10^{ - 8} } \right)$ in bovine enhancer sets

The meta-analysis recovered 92% of the significant variants from GWAS, and revealed additional variants that were not significant in the separate bull and cow GWAS (Table 3) and [see Additional file 1: Figure S1]. Significant variants were clustered on chromosomes 5, 14 and 27 for FY, chromosomes 5, 6, 14, 15, 20 for MY and chromosomes 5, 6, 11, 12, 14 and 16 for PY (Fig. 3). The Villar H3K27ac enhancer set had the largest number of significant variants (FY: 419, MY: 538, and PY: 289 variants) that spread across chromosomes 5, 6, 14, 15, 16, 20 and 27, followed by the Villar H3K4me3 set (FY: 260, MY: 273, and PY: 191 variants) that spread across chromosomes 5 and 14, dbSUPER (FY: 19, MY: 43, and PY: 19 variants) that spread across chromosomes 5, 6, 14, 15 and 20, VISTA (FY: 4, and MY: 12 variants) that spread across chromosomes 5 and 14, and FANTOM5 (FY: 3, MY: 9, and PY: 1 variants) that spread across chromosomes 14 and 15. Villar H3K27ac and dbSUPER were the two major enhancer sets that captured significant variants, with the Villar H3K27ac set covering 78% of the Villar H3K4me3 significant variants, and dbSUPER covering 50% of the VISTA and 38% of the FANTOM5 significant variants (Table 4; Fig. 4). However, the significant variants in the Villar H3K27ac and dbSUPER enhancer sets differed significantly, with less than 2% of the dbSUPER and less than 0.1% of the H3K27ac significant variants being identical (Table 4; Fig. 4).

**Table 4:** Degree of overlap between significant variants $\left( {P \le 10^{ - 8} } \right)$ in the sets of bovine enhancers analysed

	VISTA (%)	FANTOM5 (%)	dbSUPER (%)	H3K4me3 (%)	H3K27Ac (%)
VISTA	100	31.25	50	0	0
FANTOM5	38.46^a^	100	38.46	0	0
dbSUPER	9.88	6.17	100	0	1.23
H3K4me3	0	0	0	100	77.90
H3K27Ac	0	0	0.08	46.04	100

^a^Each value in the table represents the ratio, expressed as a percentage, of the total overlapping variants $\left( {P \le 10^{ - 8} } \right)$ between the two enhancer sets listed in a row and column, relative to the total number of significant variants in the enhancer set listed in the corresponding row

### Enrichment analysis

**Fig. 5:**
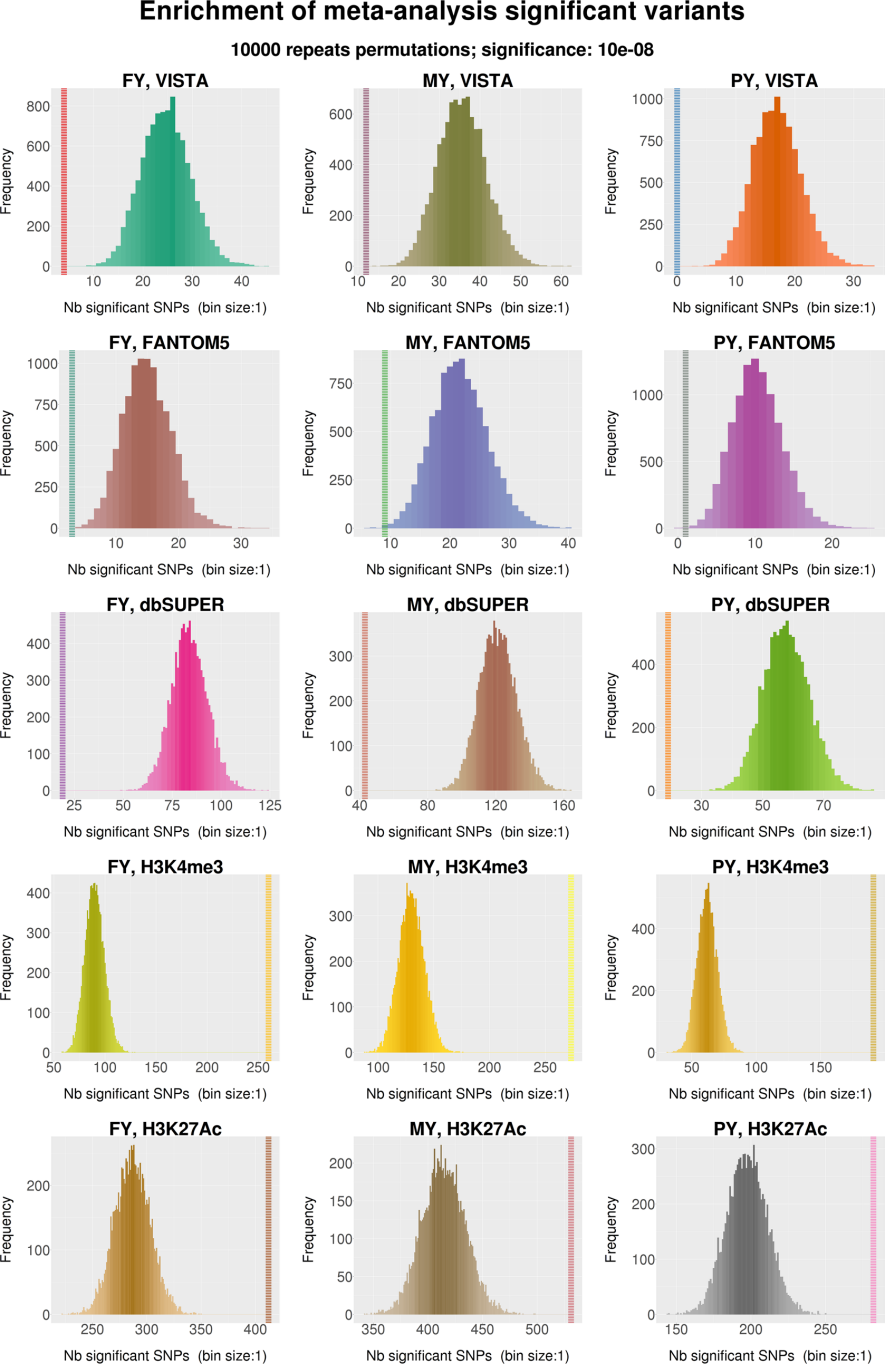
Enrichment of significant SNPs $\left( {P \le 10^{ - 8} } \right)$ in all enhancer sets. The *vertical line* indicates the number of significant variants in the original analysis. The *histograms* represent the number of significant variants in random samplings. If an analysis was significant, the vertical line would be on the *right* to the histogram and clearly separated from the histogram

A permutation test with 10,000 repeats was performed to examine the enhancer sets for their global sequence wide significance. Only the Villar H3K4me3 and H3K27ac enhancers demonstrated genome-wide significance across all phenotypes $\left( {P \le 10^{ - 8} } \right)$, whereas the homology-based enhancers did not show such a high level of enrichment in significant variants associated with milk production traits (Table 5; Fig. 5). Since dbSUPER comprised clusters of enhancers, we expanded the length of dbSUPER putative bovine enhancers, such that any sequences that were within less than 30 kb to each other were merged into a single longer enhancer sequence. The permutation test was then applied to the expanded dbSUPER enhancer sequences but the enrichment signal remained low.

**Table 5:** Enrichment of significant enhancer SNPs $\left( {P \le 10^{ - 8} } \right)$ for milk production traits in the permutation tests

Phenotype	Database	All SNPs/a subset of SNPs	Fold change^a^	Rank^b^
FY	VISTA	All	0.16235	0.0001
MY			0.336793	0.0001
PY			0	0.0001
FY	FANTOM5		0.200888	0.0001
MY			0.414773	0.0018
PY			0.096781	0.0001
FY	dbSUPER		0.226716	0.0001
MY			0.354855	0.0001
PY			0.329258	0.0001
FY	Villar (H3K4me3)		2.892337	<0.0001
MY			2.100798	<0.0001
PY			3.081699	<0.0001
FY	Villar (H3K27ac)		1.459357	<0.0001
MY			1.29579	<0.0001
PY			1.458739	<0.0001
FY	Villar:H3K4me3	H3K4me3-specific only	3.358042	<0.0001
MY			2.440737	<0.0001
PY			4.414017	<0.0001
FY	Villar:H3K27ac	H3K27ac-sepcific only	0.967677	0.3063
MY			1.023431	0.647
PY			0.953471	0.2794
FY	Villar:H3K4me3 and H3K27ac	Overlaps: H3K4me3 and H3K27ac	2.795722	<0.0001
MY			2.025759	<0.0001
PY			2.801491	<0.0001

^a^Fold change is the ratio between the actual number of significant SNPs in an enhancer set and the mean number of all significant SNPs in the 10,000 random samples

^b^Ranking position of the actual number of significant SNPs in an enhancer set within the distribution of all the numbers of significant SNPs for the 10,000 random samples; if the actual number of significant SNPs was the largest among all the numbers of the 10,000 random significant SNPs, the rank was set to <0.0001; otherwise it was denoted as the ranking position of the actual number of significant SNPs among the number of random significant SNPs

**Fig. 6:**
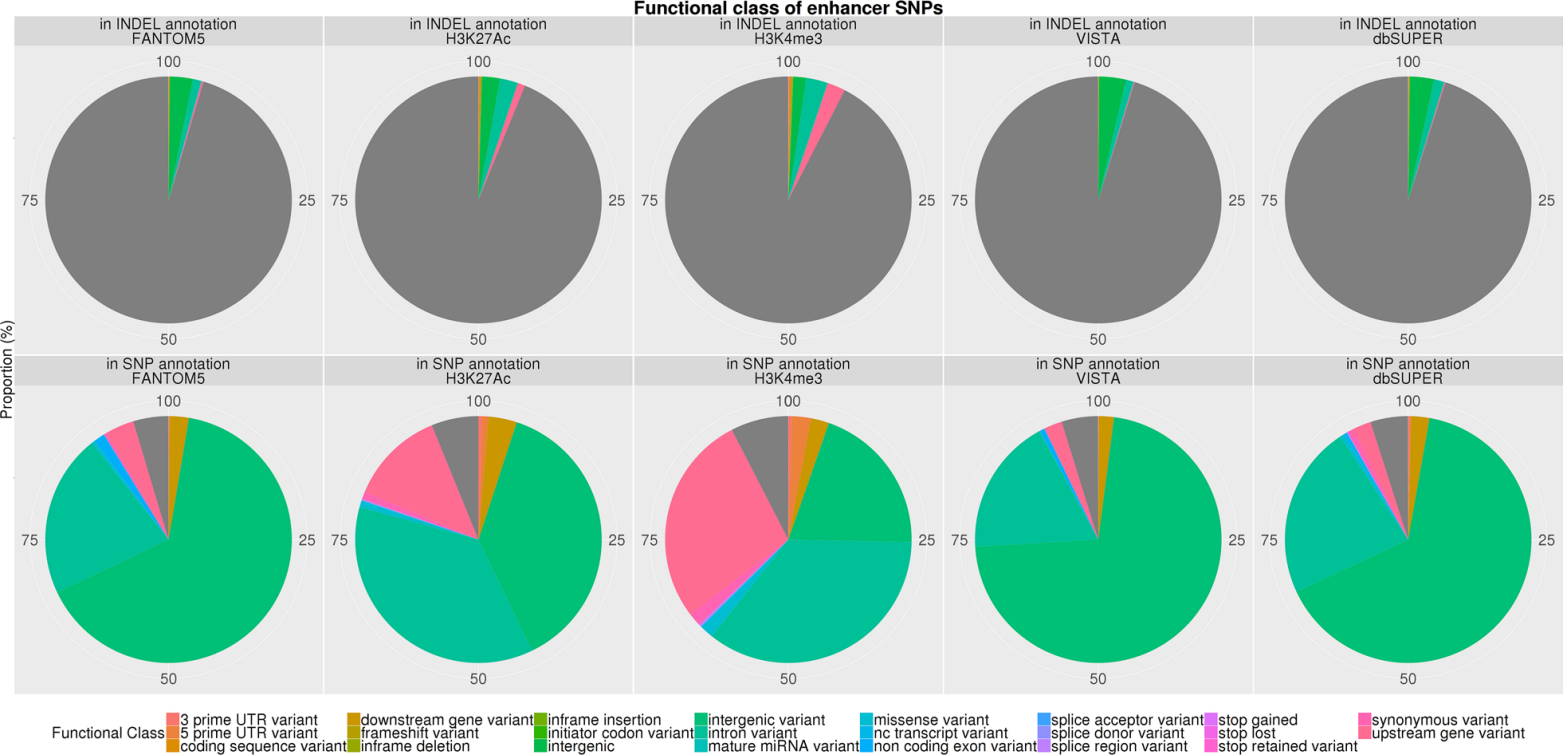
Functional class of enhancer SNPs. Each *pie chart* demonstrates the proportion of the total number of SNPs in the putative enhancer regions in the functional classes. The *grey area* represents variants without functional class annotation

**Fig. 7:**
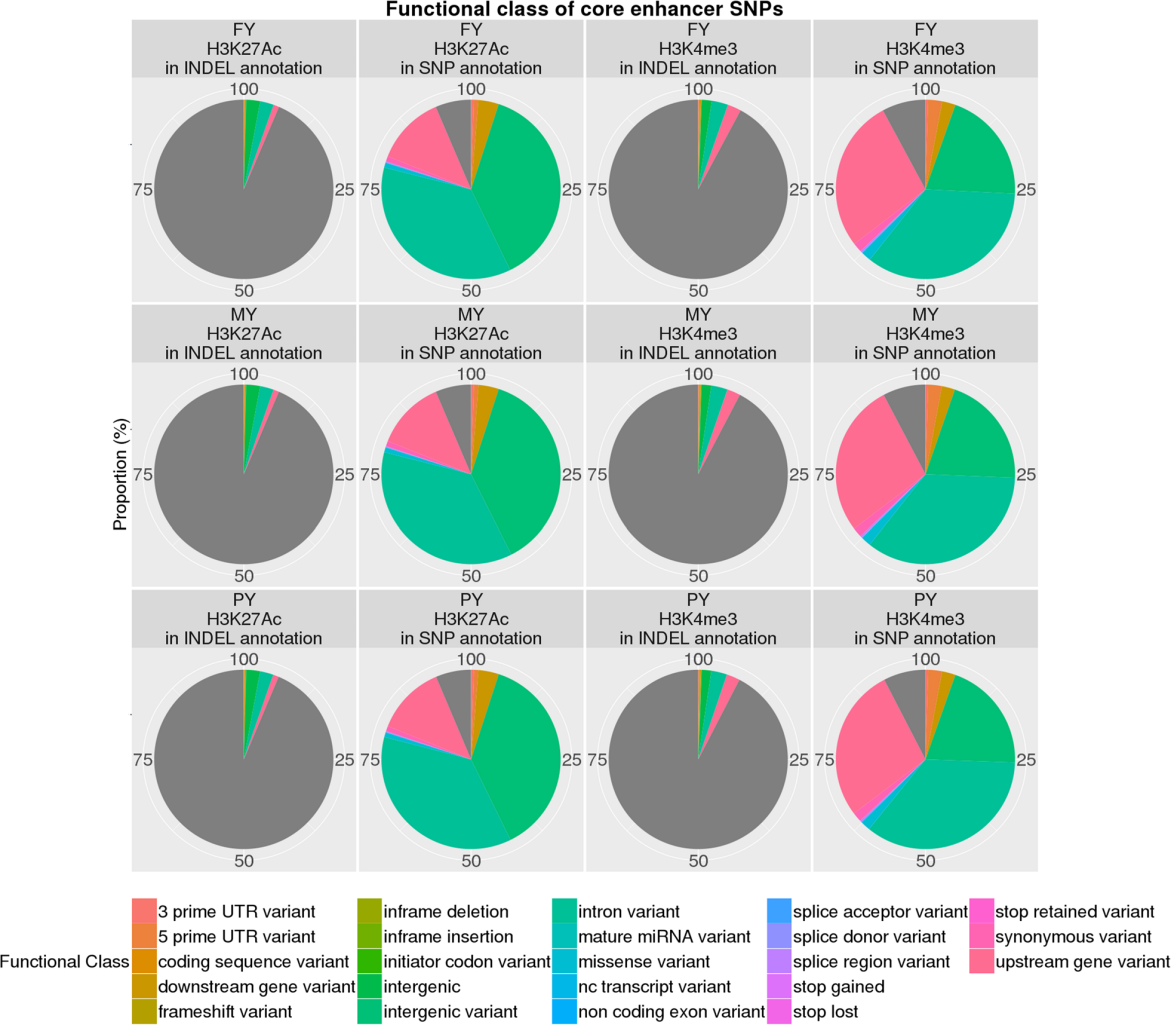
Functional class of core enhancer SNPs. Each *pie chart* demonstrates the proportion of the total number of SNPs in the Villar identified regions in the functional classes. The *grey area* represents variants without functional class annotation

Only the bovine-specific Villar H3K4me3 and H3K27ac enhancer sets demonstrated high levels of enrichment whereas homology-based enhancer sets all showed low levels of enrichment in GSEA. Around 29% of the SNPs in the Villar H3K4me3 enhancer set and 35% of the SNPs in the H3K27ac enhancer set accounted for the enrichment signals in milk production traits [see Additional file 4: Table S1]. These GSEA core enhancer SNPs were located across all the chromosomes regardless of the phenotype cohorts or histone modification signals. The number of core H3K4me3 SNPs were, in decreasing order, within intronic, upstream, intergenic, 5′-UTR, downstream, 3′-UTR, splicing, non-coding exonic and stop regions (Figs. 6, 7), whereas the core H3K27ac SNPs followed a slightly different order, i.e. within intergenic, intronic, upstream, downstream, 5′-UTR, 3′-UTR, splicing, non-coding exonic and stop regions (Figs. 6, 7).

To demonstrate the power of GSEA over the permutation test, we examined the relationship between P value threshold and SNP location. We found that the SNPs located close to a gene tended to be more significant than their counterparts in intergenic regions. Most H3K27ac-specific SNPs were intergenic and H3K4me3-specific SNPs were located in the vicinity of transcription start sites (TSS) [see Additional file 2: Figure S2 and Additional file 3: Figure S3]. As a result, the H3K4me3-specific SNPs tended to show a higher level of enrichment in the permutation test [see Additional file 4: Table S1]. However, the GSEA analysis revealed that more than 82% of the core SNPs responsible for the GSEA signal in the H3K4me3 set were also in the H3K27ac set, but more than 74% of the core SNPs in the H3K27ac set were not in the H3K4me3 set [see Additional file 5: Table S2]. This means that the H3K27ac-specific SNPs contributed some additional enrichment signal although their P values did not pass the $P \le 10^{ - 8}$ threshold.

## Discussion

The first goal of this study was to identify and improve the annotation of enhancer regions in the bovine genome. To create a library of bovine enhancers, we used publicly available human and mouse enhancer databases from VISTA, FANTOM5 and dbSUPER, along with the bovine enhancer data that were detected by ChIP-Seq from the Villar D et al. (2015) study. VISTA contains ultra-conserved developmental enhancer sequences with more than 96% of these being mapped to the bovine genome (Table 1). DbSUPER included more than 92% sequences that were mapped to the bovine genome (Table 1) probably because it contains long genomic sequences from clusters of enhancers that are closely located, which increases their chances of being mapped. The FANTOM5 data comprises very short sequences that were mapped very sparsely to the bovine genome when searched by sequence similarity in BLASTn (9.15%; Table 1) but were well recovered by liftOver which uses information from whole-genome comparisons to tolerate more frequent changes between query and target sequences (88.77%; Table 1). We exploited homologous mammalian enhancer data to identify bovine enhancers and the results are in agreement with previous findings [25, 29, 48–50] that showed that enhancer sequences, particularly the short and function-specific enhancers, are poorly conserved across species.

The second goal of this study was to validate our candidate bovine enhancer sites. We used a multi-breed GWAS followed by meta-analysis and enrichment analysis approach to examine if significant variants associated with milk production traits from meta-analyses are enriched in bovine putative enhancer sets. The genome-wide significant variants that were detected by this procedure are located in genes that affect milk production traits in cattle, in novel candidate genes, and in our candidate bovine enhancer sets. Both the permutation test and GSEA showed that only the Villar H3K4me3 and Villar H3K27ac predicted enhancer regions were significantly enriched with SNPs that are associated with the complex traits analysed here. The Villar H3K4me3 and H3K27ac enhancer sets were respectively 2.0 to 3.0-fold and 1.3 to 1.5-fold more enriched with variants that affect milk production traits than the rest of the genome (Table 5). Furthermore, the results of the permutation test and GSEA showed that the enriched H3K4me3 SNPs had significant effects within narrow genomic intervals close to genes. In addition, we observed that, in general, the H3K27ac enhancer regions encompassed the H3K4me3 enhancer regions but that most of the signals in the H3K27ac enhancer regions were located far from genes, and had small but significant effects. This finding is in line with existing literature that reports that the H3K4me3 enhancer regions display sharper peaks around TSS [51], the H3K27ac enhancer regions cover broader domains that are roughly equally distributed between intergenic and intronic regions [12], and that the proportion of SNPs at TSS reaching a significance level of −log10 (P value) higher than 10 is 50 to 100 times greater than that of SNPs in intergenic regions [52].

Our analysis did not show enrichment with enhancer regions for any production trait in any homology-based enhancer sets from VISTA, FANTOM5 and dbSUPER. There are two possible reasons for this finding. First, none of the VISTA, FANTOM5 and dbSUPER enhancer sets were sampled from a tissue that is directly linked to milk production (an example of tissue that is directly linked to milk production is the lactating mammary gland tissue). Therefore, the homology-based enhancers that are relevant to milk production may not be present in our downloaded databases and therefore cannot be considered in this study. Second, although VISTA, FANTOM5 and dbSUPER may contain sets from tissues that are involved in the physiological processes that are fundamental for the regulation of milk production, the procedure to map these sequences to the bovine genome is based on the identification of conserved sequences with human and mouse sequences, and as a result, the bovine-specific mutations within the homology-based enhancers cannot be captured [53]. Our results support the hypothesis of a rapid evolution of the enhancer sequences since the bovine-specific liver enhancer regions differed substantially from all homology-based liver enhancer regions (Fig. 2), which suggests that bovine-specific enhancers are more likely to be enriched with causative mutations that affect complex traits, in this case milk production. Our results, combined with the above reasons, highlight the complexity of the genomic regulatory machinery and the importance of analysing enhancers specific to the species under investigation [4]. The success of this study based on regulatory landscape data from one tissue type (liver) using two epigenetic marks (H3K4me3 and H3K27ac) indicates that our results might be even more convincing if we had data from more tissue types.

On chromosome 14, the observed enrichment signal in enhancer regions could be due to SNPs in linkage disequilibrium (LD) with the well-known mutation in the *DGAT1* gene [54]. To account for LD confounding around the *DGAT1* mutation, we re-ran our GWAS on chromosome 14 by correcting for the effect of the *DGAT1* gene by including the causative mutation in the model as a fixed effect. The correlations of the SNP effects (P values) between before and after the correction were 85% (59%), which showed that there were other significant SNPs on chromosome 14 apart from the *DGAT1* mutation. After correction, no significant SNPs remained in the VISTA and FANTOM5 enhancer sets for all milk production traits, but 34 to 67% significant SNPs remained in the Villar H3K4me3, Villar H3K27ac and dbSUPER enhancer sets [see Additional file 6: Table S3]. The SNPs that remained significant after the correction in the putative enhancer sets were located in regions up to 10 Mb around the *DGAT1* gene. In addition, while the Villar H3K4me3 and dbSUPER enhancer sets had no corrected significant variants within the *DGAT1* gene, the Villar H3K27ac enhancer set include one such significant variant (Chr14: 1797137 in FY and MY cohorts) in the first intron of the *DGAT1* gene.

Several candidate regulatory variants that affect the expression of *MGST1* have been reported to be responsible for the QTL effect on chromosome 5 for milk production traits [38, 55]. We found that they were within or close to the Villar H3K4me3 and H3K27ac enhancer regions, which provided evidence that the causal mutation is in fact a regulatory variant [see Additional file 7: Table S4 and Additional file 8: Figure S4].

Several studies have reported that the variant Chr6:88741762 is significantly associated with milk production traits [38]. This variant was significant in both our MY and PY cohorts, within the H3K27ac set, and is located 2470 bp upstream of the *GC* gene. An RNA-Seq analysis [56] showed that *GC* was most highly expressed in the liver and over-expressed in the mammary gland, and that there was a strong allele-specific expression in liver compared to 17 other bovine tissues [see Additional file 8: Figure S4].

## Conclusions

This study used mammalian enhancer prediction data and bovine trait association to provide a functional variance analysis of candidate bovine enhancer regions. Overall, our findings agree with previous research that enhancer sequences are species-specific and rarely conserved across species. We conclude that bovine-specific histone data such as H3K4me3 and H3K27ac are essential for the successful functional annotation of bovine enhancer regions. Although the amount of bovine enhancer information is limited, we have successfully identified many genomic regions as potential enhancers and demonstrated that variation in these regions is associated with variation in animal production traits. Future studies will benefit from the combination of information from topological domain association, expression quantitative trait loci and bovine ChIP-Seq data, such as that generated from the Functional Annotation of Animal Genomes (FAANG) consortium [57], to accelerate the identification of mutations that affect complex traits.

## Additional files

**Additional file 1: Figure S1.** Comparison of the similarity between GWAS significant SNPs and meta-analysis significant SNPs (*P* ≤ 10^−8^). A meta-analysis significant variant was counted twice, one for the bull and one for the cow to account for the identity of a GWAS significant variant.

**Additional file 2: Figure S2.** Comparison of the significant SNPs (*P* ≤ 10^−8^) between the Villar2015 H3K27ac and H3K4me3 enhancer sets.

**Additional file 3: Figure S3.** Comparison of the functional class of significant SNPs (*P* ≤ 10^−8^) between the bovine liver H3K4me3 and H3K27ac enhancer sets.

**Additional file 4: Table S1.** Properties of GSEA core SNPs in the Villar (H3K4me3 and H3K27ac) enhancer sets. The proportion of significant GSEA core SNPs in the H3K27ac set doubled when the P value threshold decreased from 10^−8^ to 10^−5^ whereas the proportion of GSEA core SNPs in the H3K4me3 set did not increase as much.

**Additional file 5: Table S2.** Proportion of GSEA core SNPs in the H3K4me3 set that are also in the H3K27ac set, and proportion of the GSEA core SNPs in the H3K27ac set that are also in the H3K4me3 set.

**Additional file 6: Table S3.** Number of significant enhancer SNPs (*P* ≤ 10^−8^) that remained on chromosome 14 after correcting for the effect of the well-known *DGAT1* mutation.

**Additional file 7: Table S4.** SNPs highlighted in previous studies [38, 55] that were also our GSEA core SNPs in the Villar (H3K4me3 and H3K7ac) sets.

**Additional file 8: Figure S4.** Manhattan plot showing the enhancer signals around the *MGST1* and *GC* genes.
