# Supplementary figures and images for: Putative enhancer sites in the bovine genome are enriched with variants affecting complex traits
Source: Genet Sel Evol. 2017 Jul 6;49:56. doi: 10.1186/s12711-017-0331-4 (PMC5499214; doi:10.1186/s12711-017-0331-4)

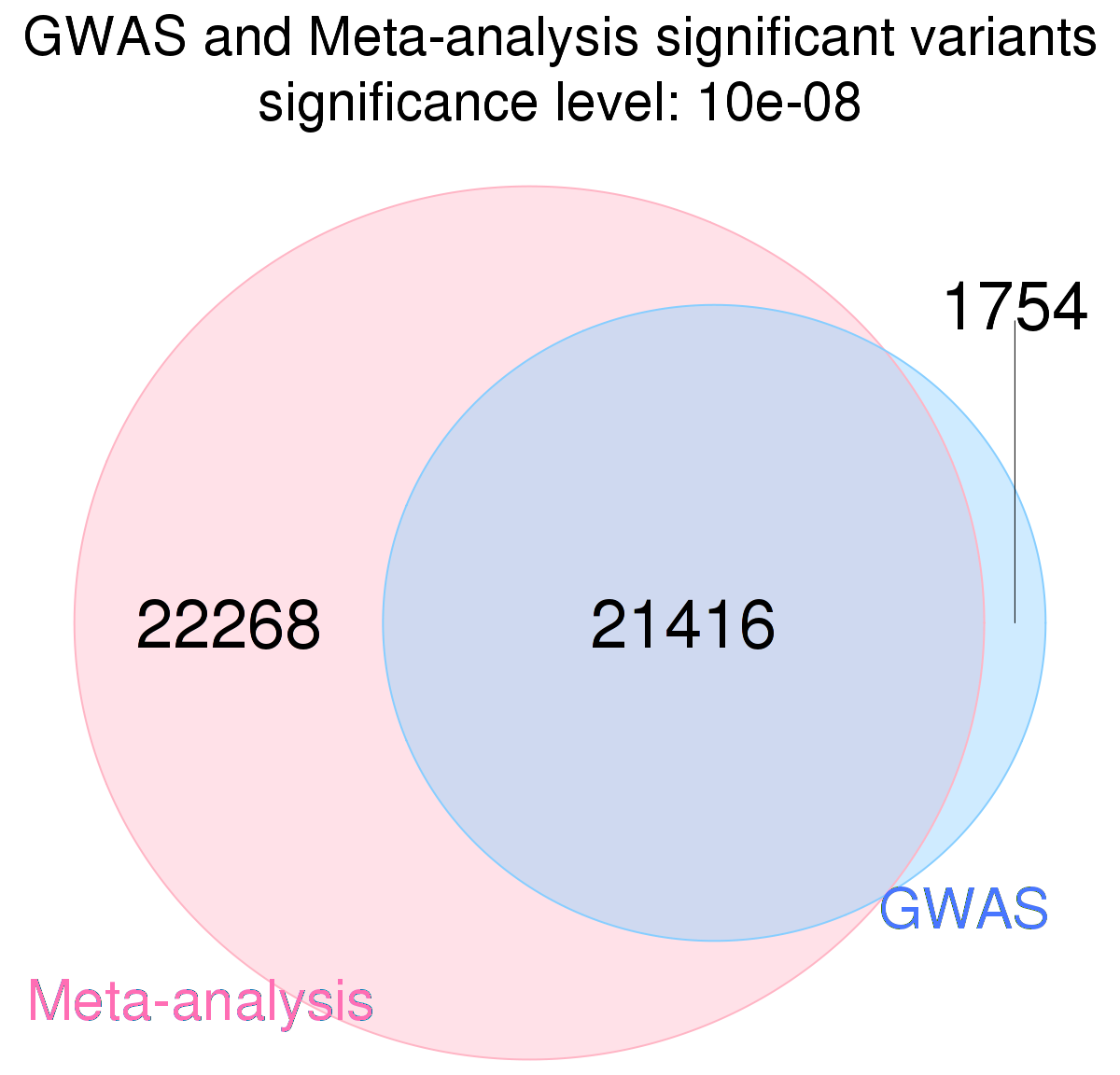

Supplement: Supplementary file 1 — Additional file 1: Figure S1. Comparison of the similarity between GWAS significant SNPs and meta-analysis significant SNPs (P ≤ 10−8). A meta-analysis significant variant was counted twice, one for the bull and one for the cow to account for the identity of a GWAS significant variant. [file 12711_2017_331_MOESM1_ESM.png]

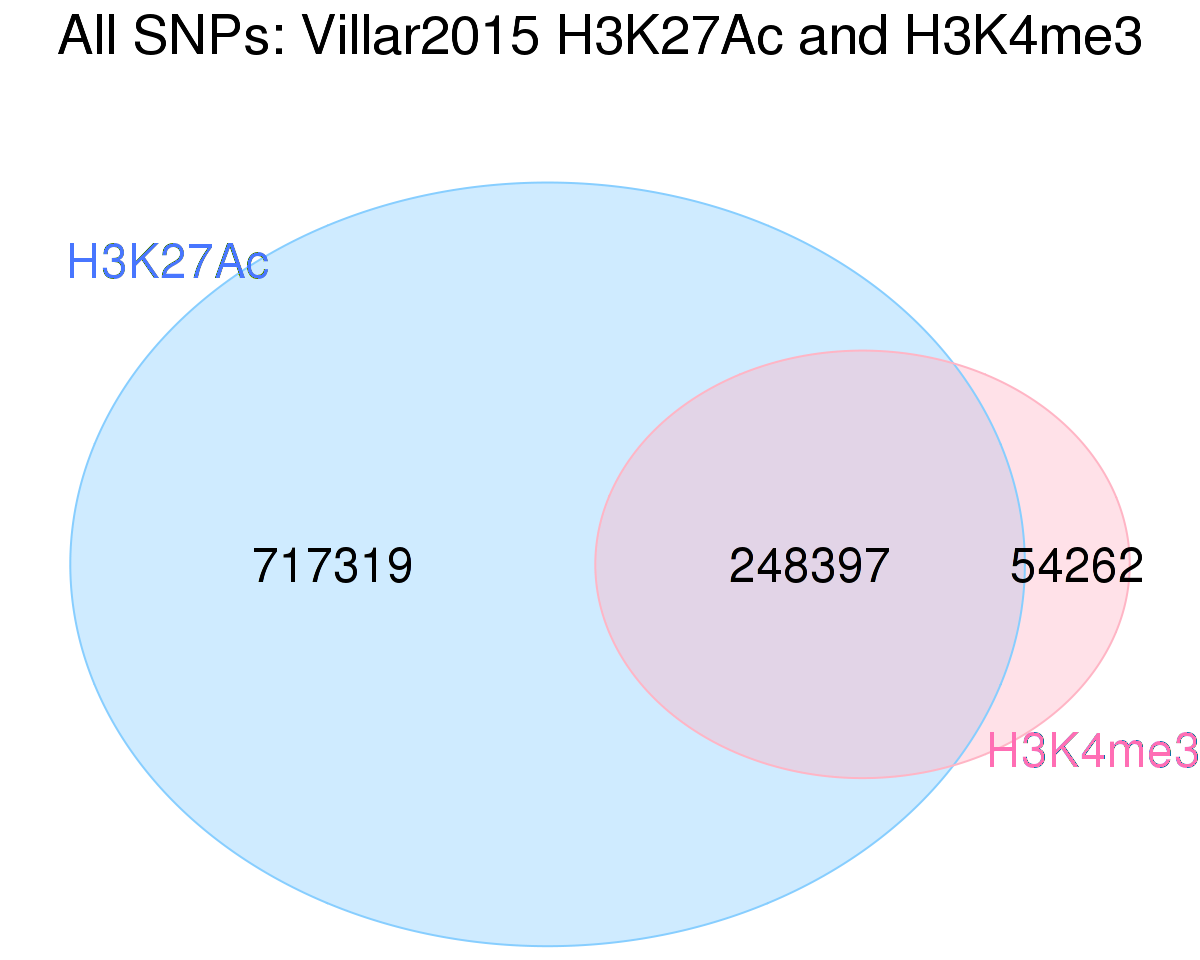

Supplement: Supplementary file 2 — Additional file 2: Figure S2. Comparison of the significant SNPs (P ≤ 10−8) between the Villar2015 H3K27ac and H3K4me3 enhancer sets. [file 12711_2017_331_MOESM2_ESM.png]

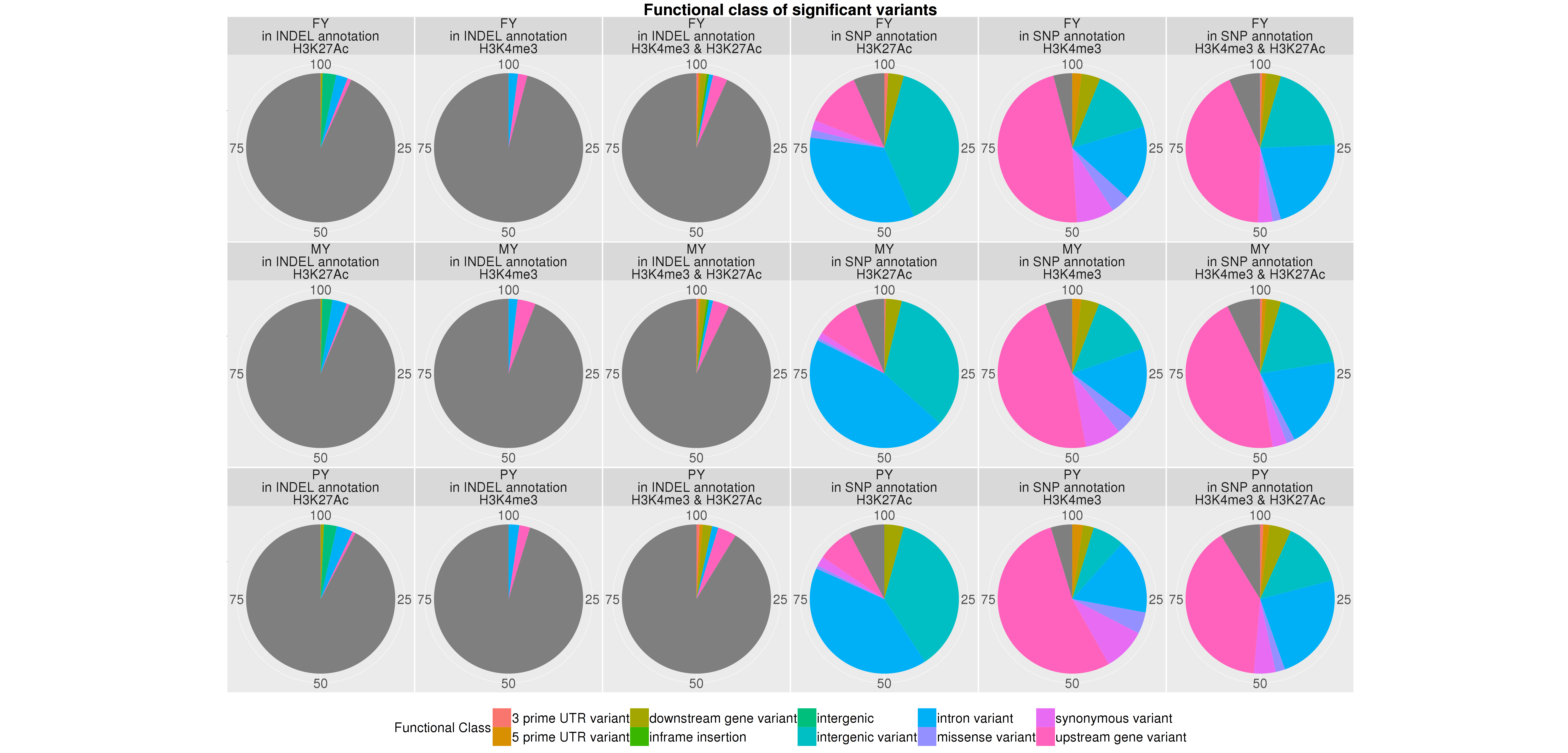

Supplement: Supplementary file 3 — Additional file 3: Figure S3. Comparison of the functional class of significant SNPs (P ≤ 10−8) between the bovine liver H3K4me3 and H3K27ac enhancer sets. [file 12711_2017_331_MOESM3_ESM.png]
